# Animations, videos and 3D models for teaching space-group symmetry

**DOI:** 10.1107/S1600576724008872

**Published:** 2024-10-16

**Authors:** Lauro Bucio, Rosario Moreno-Tovar, Edilberto Hernández-Juárez, Andrea S. Sandoval-Santiago, Nerith R. Elejalde-Cadena, Andrés Bucio, Moises Falcón-Moreno, Ivonne Rosales-Chávez

**Affiliations:** a Laboratorio de Cristalofísica y Materiales Naturales, Instituto de Física Universidad Nacional Autónoma de México Circuito de la Investigación Científica s/n, Ciudad Universitaria, Coyoacán 04510Mexico City Mexico; b Independent Researcher, Guadalajara, Jalisco, Mexico; c Departamento de Física y Química Teórica, Facultad de Química Universidad Nacional Autónoma de México Ciudad Universitaria, Coyoacán Mexico City Mexico; d Laboratorio Nacional de Vivienda y Comunidades Sustentables, LNVCS, Facultad de Arquitectura Universidad Nacional Autonoma de Mexico 04519Mexico City Mexico; Wilfrid Laurier University, Waterloo, Ontario, Canada

**Keywords:** animations, videos, 3D models, space groups, Seitz operator, symmetry operations

## Abstract

A series of animations, videos and 3D models that were developed, filmed or built to teach the symmetry properties of crystals are described. At first, these resources were designed for graduate students taking a basic crystallography course, coming from different careers, at the National Autonomous University of Mexico. However, the COVID-19 pandemic had the effect of accelerating the generation of didactic material. Besides our experience with postgraduate students, we have noted that 3D models attract the attention of children, and therefore we believe that these models are particularly useful for teaching children about the assembled arrangements of crystal structures.

## Abstract 2

Animations, videos and 3D models have been designed to visualize the effects of symmetry operators on selected cases of crystal structures, pointing out the relationship with the diagrams published in *International Tables for Crystallography*, Vol. A.

## 1. Introduction

The material organization of signs, diagrams and symbols has had a great impact in the production of scientific knowledge (Francoeur, 2001 ▸). For point- and space-group symmetry and their diagrammatic representation, the closer cooperation that started in 1929 between crystallographers at an international level gave rise to the preparation of standardized space-group tables and crystallographic nomenclature (Brock, 2014 ▸). The latest editions of *International Tables for Crystallography*, Vol. A (*IT*A, 2005 ▸, 2016 ▸), are of significant relevance for transmitting information on symmetry.

In the early years of crystal structure determination, Buerger & Butler (1936 ▸) pointed out that structural data in the form of cell dimensions, space group and atomic coordinates are of very little use if someone desires correct visual pictures of the crystal structures. At that time, several publications were devoted to methodologies for modelling crystal structures using wires and commercially available wooden spheres of different sizes. Thereby, it was possible to build a model such that 1 Å = 2 inches (Wyckoff & Ksanda, 1926 ▸; Buerger & Butler, 1936 ▸; Buerger & Butler, 1938 ▸; Dorris *et al.*, 1938 ▸; Seymour, 1938 ▸; Wooster & Knott, 1938 ▸). Several researchers were devoted to the study of the structure of minerals and other inorganic crystals by X-ray diffraction, and accurate values of interatomic distances in crystals were obtained. The necessity of developing a system of radii for atoms that explained the interatomic distances measured in inorganic substances emerged (Pauling, 2015 ▸). As a result, a set of atomic radii were available to anyone who wanted to build 3D models for crystal structures.

In later years, Pauling considered the importance of having information about interatomic distances, bond angles and other configurational parameters in order to obtain a reliable prediction of reasonable configurations for polypeptide chains. This would have an impact on the study of the structure of both fibrous and globular proteins, as well as of synthetic polypeptides (Pauling *et al.*, 1951 ▸). Historically, a crucial starting point was the complete and precise determination of the crystal structures of amino acids, peptides and other simple organic substances. The first was hexa­methyl­ene tetramine by Dickinson & Raymond (1923 ▸), which forms crystals with cubic symmetry. Bond lengths and bond angles were extracted with success by the method of electron diffraction (Pauling, 2015 ▸). Determinations of amino acid and peptide structures and the understanding of protein structures were greatly facilitated using accurately constructed 3D models. Interestingly, a technical description of the models used at Caltech (USA) pointed out that they were intended as ‘substitutes for calculations in investigations of the probable configuration of the protein chain in proteins’ (Corey & Pauling, 1953 ▸). Two types of model emerged, being complementary and of particular importance: the space-filling and the skeletal (Walton, 1978 ▸). The former had the advantage of examining fundamental questions such as steric hindrance (Francoeur, 2001 ▸) and the second allowed a connection to be made between the atoms in a molecule and its arrangement. The work of Pauling and Corey on the polypeptide chain would eventually have strong influence when the structure of DNA was revealed (Watson & Crick, 1953 ▸; Watson, 1968 ▸).

In the middle of the last century, Bragg & Nye (1947 ▸) published an extraordinary model consisting of an assemblage of bubbles representing the crystal structure of a metal. With this model, they were able to simulate observed effects, such as grain boundaries, dislocations and other types of fault. Since then, ingenious methodologies for representing crystal lattices, symmetry, crystal structures and their properties have been documented (Buseck, 1970 ▸; Kantardjieff *et al.*, 2010 ▸; Sunderland, 2014 ▸; Lenzer *et al.*, 2019 ▸; Murray *et al.*, 2024 ▸). The ability to visualize and manipulate crystal structures has been crucial for understanding different structural types and formations. Interactive visualization has largely displaced molecular modelling in the study of crystal structures (Driessen *et al.*, 1988 ▸; Alves *et al.*, 2021 ▸). Software resources have included crystallization phenomenon analysis (Chadwick *et al.*, 2019 ▸), allowing the display of crystal structures, as well as the prediction or display of crystal morphologies such as in *Mercury* (Macrae *et al.*, 2020 ▸) or *KrystalShaper* (Weber, 2020 ▸). *KrystalShaper* allows one to obtain and print polyhedral nets to build paper models of crystal shapes. Very helpful software has been developed for visualizing inorganic or mineral structures, such as *VESTA* (Momma & Izumi, 2011 ▸), and other software such as *DIAMOND* (Pennington, 1999 ▸) and *ChimeraX* (Pettersen *et al.*, 2021 ▸); this last was designed for interactive visualization from the UCSF Resource for Biocomputing, Visualization and Informatics (University of California San Francisco, USA). *PowderCell* (Kraus & Nolze, 1996 ▸) allows the display of crystal structures and the calculation of powder diffractograms, and offers non-conventional features like the generation of subgroups or representation of a crystal structure using non-standard settings for a given space-group type (Nolze, 2002 ▸). *Jmol* (Hanson, 2010 ▸) highlights the essential characteristics of unit cells, space groups and symmetry operators; it has been used for teaching symmetry and for space-group visualization (*e.g.*https://symotter.org).

The above illustrate how the development of 3D models and access to computing software have played an important role in the rise of crystallography and the understanding of crystal structures. Further, to understand how to describe the symmetry properties of a crystal structure by means of the symmetry operations of its space group, it is extremely important to adhere to the symbols, diagrams and nomenclature used in *International Tables for Crystallography* (*IT*A, 2005 ▸, 2016 ▸). Some of the specific references related to this matter are Burns & Glazer (2013 ▸), Aroyo *et al.* (2006 ▸), Dauter & Jaskolski (2010 ▸) and Nespolo *et al.* (2018 ▸), among others.

Additionally, there are many web sites containing diverse interesting resources. These include among others the Bilbao Crystallographic Server, which offers free of charge its crystallographic and solid-state programs and utilities (Aroyo *et al.*, 2011 ▸; https://www.cryst.ehu.es); a tutorial on symmetry and space groups by Jasinsky & Foxman (Foxman, 2021 ▸; https://sites.google.com/brandeis.edu/foxman-group/teaching/space-groups) with links to consult historical, professional and pedagogic topics; and course material on symmetry teaching and space groups with online animation and videos by Hoffmann (https://crystalsymmetry.wordpress.com).

The purpose of the present contribution is to show how animations and videos in the form of a few short briefing capsules, as well as 3D models, can be useful to help in visualizing the symmetry properties of a crystal, following the diagrams and symbols used in *IT*A. The topics and distinctive features we have considered for the elaboration of the short briefing capsules can be summarized as follows:

(i) *Models*. Crystalline structures with appropriate pedagogical qualities were considered to illustrate their symmetry properties, although certain aspects with practical relevance such as use and applications were also pondered.

(ii) *Asymmetric unit*. All asymmetric units great and small were contemplated to show the symmetry properties of crystal structures, from very small such as the case of five atoms for the crystal structure of ice, to hundreds of atoms for the case of insulin.

(iii) *Matrix representation for rotational symmetry operations*. Under the (**a**, **b**, **c**) vectorial basis, the animated movement resulting from the linear mapping of a rotational symmetry operation (isometry) is described, allowing its matrix representation through geometric reasoning.

(iv) *Seitz symbols for a symmetry operation*. With the application of each symmetry operation on the asymmetric unit, both rotational (matrix) and translational parts of the Seitz operator are exposed in the animation, showing how the 3D space is occupied by isometric mapping.

(v) *Diagrams for representing general positions and symmetry elements*. In the animations, when a Seitz operator is applied to the asymmetric unit of the crystal, the portion of atoms generated by the symmetry is shown simultaneously, together with the corresponding position in the general positions diagram. With the information obtained from the general positions generated by each Seitz operator, the diagram of symmetry elements is constructed by means of animations.

(vi) *Centred lattices*. The use of the Seitz operators and how the information is organized in *IT*A for the general coordinates and diagrams of symmetry elements is described for the case of centred lattices, such as occur for the crystal structures of insulin and cocoa butter.

(vii) *Relocation of symmetry elements*. The combination of rotational symmetry (proper or improper) with translations is reinterpreted in some of the animations in terms of relocation of the symmetry elements.

(viii) *Screw axes and glide planes*. Symmetry operations such as screw axes and glide planes, represented through the Seitz symbols, are described in the animations, giving them a geometric sense in selected crystal structures such as ice or cocoa butter. Frequently ‘roto-translation’ symmetry requires reinterpretation as screw axes or glide planes, with or without relocation of symmetry elements in the 3D space.

(ix) *Coset decomposition of a space group*. A given space group can be represented by a left coset decomposition with respect to its translation subgroup. For this case, polymorph V of cocoa butter was selected for its simplicity, ease of animation and small number of coset representatives, and as a good and interesting example of a centred lattice. Each coset, headed by a *coset representative*, has been associated in the crystal structure with a single specific colour.

(x) *Running time*. The set of designed briefing capsules have short running times, around 1½ min for the shortest and around 31 min for the longest.

With the design described above – addressed in the first instance to graduate students as a support to lectures given in a classroom – we set out to facilitate understanding and the posing of questions related to the type of packing for a crystalline arrangement, the nature of interactions between atoms or molecules, the rate of growth in certain directions and the reason for certain symmetry properties, among many others.

## 2. Materials and methods

### 2.1. Models for crystal structures

The six crystalline materials listed in Table 1 ▸ were selected to illustrate their symmetry properties since we found useful pedagogical qualities in them. In addition, they cover almost all the crystal systems, comprise primitive and centred lattices (involving the rhombohedral centred space group described with hexagonal axes), encompass a wide range of symmetry operations and diverse sizes for the asymmetric unit, and are miscellaneous types of compound with varied uses and significance for humans.

With the crystal structures listed in Table 1 ▸, we intend to explain different topics of space-group symmetry.

#### 2.1.1. Lupeol

The first crystal structure is lupeol, which is an excellent case for illustrating the action of symmetry operations on the asymmetric unit. The asymmetric unit consists of one single molecule of lupeol, where any symmetry operation of the space group acts on all and each one of the atomic positions creating an identical molecule of lupeol, equivalent by symmetry to the asymmetric unit. For the description of its space group only four symmetry operations are needed, and the crystal system is tetragonal, which is not so difficult to visualize. Lupeol belongs to a group of substances known as *secondary metabolites*, which are of extreme importance for understanding the mechanisms of plant interactions with their environment and their relationships with other living organisms.

#### 2.1.2. Insulin

An insulin dimer is an example of a ‘giant asymmetric unit’ that can be found in a crystal structure. As a macromolecule, the insulin dimer is structurally very complex. Three dimeric units related by a threefold symmetry axis assemble to form a hexamer in the shape of an oblate spheroid. The spheroids pack together as sheets of spheres arranged at the vertices of a triangular net. The sheets are stacked upon one another in such a way that every third sheet eclipses the first one of the sequence of three. Insulin is a hormone important for regulating glucose levels in blood, and it exists in the body stored as a stable inactive hexameric form in the pancreas. When insulin is secreted by the pancreas in response to an increase in glucose levels in the blood, the insulin hexamer dissociates to dimers (breaking the threefold symmetry) and then to monomers, which are biologically active in promoting glucose metabolism.

#### 2.1.3. Ice

Unlike the cases of the previous crystal structures, in the crystal structure of ice the water molecule has internal symmetry given by its point group *C*_2*v*_ (*mm*2). Some of the symmetry operations are included among those of its space group. The arrangement of water molecules in ice is attractive for visually demonstrating the presence of diverse symmetry properties such as rotation axes, mirror planes, glide planes and screw axes of symmetry. On the other hand, the crystal structure of ice presents structural disorder that is concerned with its diverse polymorphic phases. Ice has had a deep and multifaceted impact on life on Earth, from climate regulation to habitat and preservation of natural resources; it has played an essential role in the ecological balance and in life on Earth as we know it.

#### 2.1.4. Aspirin

In this case, the asymmetric unit consists of an aspirin molecule, which has no internal symmetry (like lupeol and insulin). This crystalline structure is ideal for demonstrating detailed symmetry operations such as inversion, screw axes and glide planes.

On the other hand, with this example, we seek to visualize crystal growth in terms of mappings by symmetry operations that give rise to the fragments that are added during crystal formation. The crystal structure and growth of aspirin crystals are of fundamental importance for understanding its solubility, stability and processability, and ultimately its efficacy as a drug for reducing blood coagulation and as a non-steroidal anti-inflammatory drug.

#### 2.1.5. Cocoa butter

Cocoa butter has at least six polymorphic forms with different melting points and different stabilities and textures. The space group that corresponds to the crystal structure of polymorph V (*C*1*c*1) has only four symmetry operations listed in *IT*A. This case is appropriate for dealing with the study of centred lattices and for understanding the left coset decomposition of a space group with respect to its translation subgroup. The concept of decomposing a space group in terms of cosets can be readily generalized from this simple example. Polymorph V of cocoa butter is crucial for the manufacture of chocolate. An in­adequate structural arrangement can lead to a ‘fat-bloom’ phenomenon, resulting in the formation of Polymorph VI, which recrystallizes on the chocolate surface, affecting its texture, appearance and sensory properties.

#### 2.1.6. Chromium potassium alum

This is a salt of chromium and potassium which has cubic symmetry. In this special case, the set of symmetry operations (necessary to build a 3D structure) can easily be used to create a mapping for the gradual generation of fragments of the crystalline solid following a macroscopically dictated pattern of crystal growth, as observed experimentally. Chromium potassium alum is a mordant important in the textile industry, used to fix dyes in textile fibres. It is also used as a chemical reaction catalyzer and as a flocculant in water-treatment processes.

### 2.2. Software

The Crystallographic Information Framework (CIF) files for lupeol, aspirin, cocoa butter and chromium potassium alum were processed using *Mercury* (Macrae *et al.*, 2020 ▸), while *VESTA* (Momma & Izumi, 2011 ▸) was used for modelling the crystal structures of ice and chromium potassium alum. *VESTA* was also helpful for displaying the crystal shapes of aspirin and chromium potassium alum after careful analysis of the images obtained from crystal growth experiments. The movements of all the images, text effects, colour changes *etc.* were performed using *PowerPoint* utilities (Microsoft, Version 16.85). At the end, .gif and .mp4 files were produced. In some cases, the final files were edited by *Filmora* (Wondershare, Version 13.3.9) to improve the presentation.

### 2.3. Crystal growth

To grow chromium potassium alum crystals, the powdered substance (4.8 g; Farmacia Paris, SA de CV, Mexico City) was dissolved in water (20 ml). Complete dissolution was ensured by heating the water using an electric grill hotplate for 2 min to ensure a completely homogeneous solution. The solution was poured onto a 90 × 15 mm Petri plate and a record of the growth was taken.

For aspirin, acetyl­salicylic acid powder (0.9931 g; Sigma–Aldrich, 99.0%) was dissolved in ethanol (20 ml). The solution was poured onto a Petri plate for recording the growth of crystals.

### 2.4. Image recording

A digital camera (Canon EOS Rebel SL3) with an EF 100 mm f/2.8 L Macro IS USM lens was used for obtaining images of the crystals growing. The camera was controlled by Python code to shoot each specified period Δ*t* (30 s). The images with the crystals growing were processed to make a video file. This was done for aspirin and chromium potassium alum.

### 2.5. Three-dimensional models

The CIF file for ice (Table 1 ▸) was used in *VESTA* to obtain the geometry and dimensions for a water molecule. *VESTA* allows export of .stl files which are appropriate for 3D printing. Several water molecules were printed using polylactic acid (PLA) as printing material. Round magnets (7 mm diameter, 2 mm thick) were embedded inside holes that were created by drilling the models. Two magnets were placed in each oxygen atom in such a way that the two north poles were exposed at two spots on its surface, while one magnet was placed in each hydrogen atom with its south pole at one spot on the surface. With the magnets correctly oriented, a correct mimic of ‘hydrogen-bonding interactions’ by the resulting magnetic forces was achieved. The CIF file for cocoa butter (Table 1 ▸) was processed by *Mercury* to display the molecule that represents the asymmetric unit, and an .stl file was exported from *Mercury* for 3D printing of a whole molecule. The space-filling style was selected to represent the molecule (*i.e.* assigning van der Waals radii to all the atoms).

## 3. Representing a space-group symmetry operation: the Seitz operator

The Seitz operator, introduced by Seitz (1935 ▸), is symbolized as {***R***|***v***}, where ***R*** represents the rotation part and ***v*** contains the components of the translation part of the symmetry operation. ***R*** can be specified by the proper or improper rotation symbol (1, 2, 3, 4, 6 or 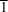, *m*, 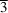, 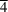, 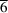), together with a superscript + or − to specify the sense of rotation (in case of ambiguity) and with a subscript consisting of three integers associated with the direction of the axis of rotation, or the normal to a plane of reflection. For ***v***, the three components of the translation part are directly placed separated by commas. When the three components are equal to zero, only one zero is placed for ***v***. The details of the nomenclature followed for ***R*** and ***v*** are described by Glazer *et al.* (2014 ▸). If the coordinates of a position vector are given by **r** = *x***a** + *y***b** + *z***c**, then the motion executed by the symmetry operation {***R***|***v***}, results in the new position vector 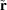 given by
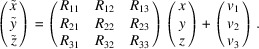


## 4. Short briefing capsules

The learning activities contained in the capsules are listed in Table 2 ▸ , along with the names of the corresponding files in the supporting information and their running times.

The use of the capsules described in Table 2 ▸ is designed to complement theoretical courses in curricula that address the symmetry described by the space groups, the notation used, diagrams, symbols *etc.* The capsules are videos that can be used individually or in groups. However, it seems that the maximum benefit can be achieved by watching the video and controlling the speed of display, rewinding, pausing or replaying scenes at will. There are capsules that are very short and can be repeated as many times as necessary. There are details in the video image field which can be appreciated after several repetitions. One of the problems that remains to be solved in the elaboration of the capsules is the optimization of the speed at which the animations move and their correct positioning in the video image field. This requires synchronization with the response of the human brain to the stimulus given by the video information to obtain a perception of knowledge that goes beyond the simple visual sensation.

In the case of the use of the capsules in a group or classroom setting by an instructor, it could be useful if, together with the blackboard where the theoretical basis of a given topic is presented, there is a screen at the side of the blackboard and, from time to time, the instructor uses some part of the capsules, showing certain parts, pausing, going backwards or whatever is desired.

### 4.1. Matrix representation for rotational symmetry operations

**Figure 1:**
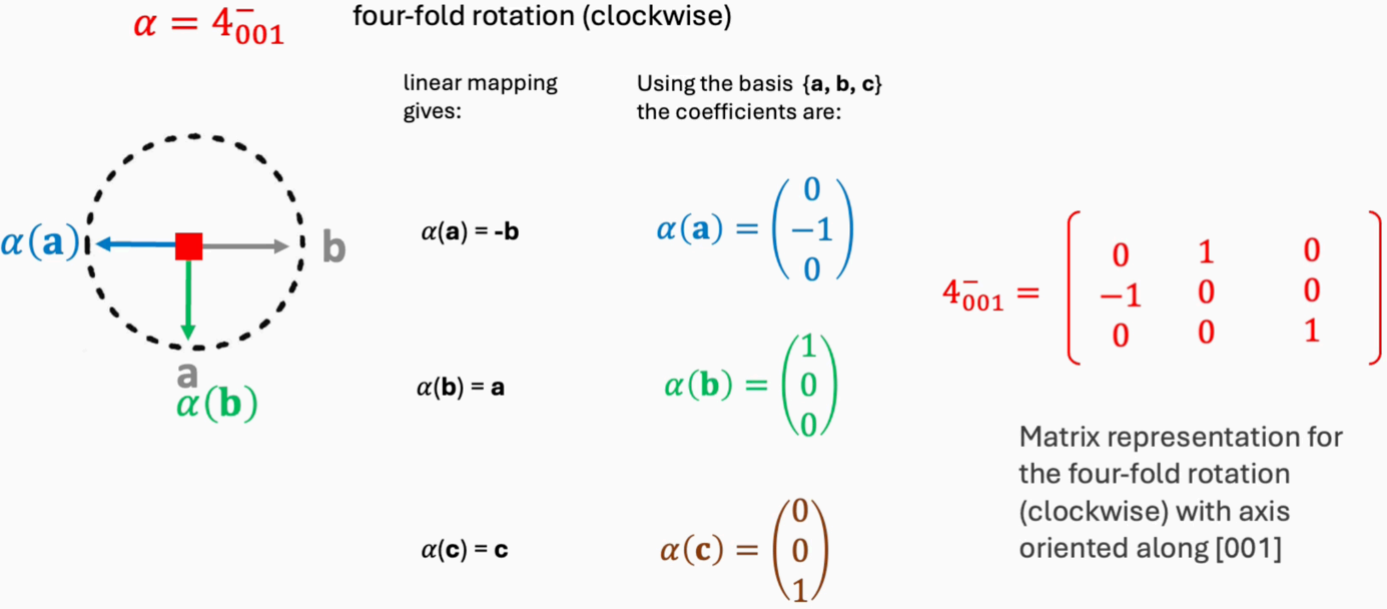
In this figure, the matrix representation for α = 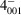 is the matrix where the first column has as entries the linear mapping coefficients of α(**a**), the second column the coefficients of α(**b**) and the third column the coefficients of α(**c**). In all these cases, the representations are with respect to the (**a**, **b**, **c**) basis.

The matrix representation for the rotational part ***R*** with respect to (**a**, **b**, **c**) can be obtained by considering the operation ***R*** as a linear mapping α which is applied to the vectors **a**, **b**, **c**, obtaining α(**a**), α(**b**), α(**c**) and the coefficients of linear mappings with respect to (**a**, **b**, **c**) as follows (Boisen & Gibbs, 2018 ▸):
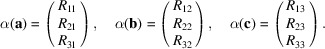
The case for 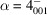 (fourfold rotation clockwise with the symmetry axis along [001]) is illustrated in Fig. 1 ▸.

The matrix representations for five isometries, α = 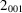, 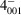, 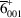, 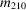 and 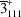, are geometrically rationalized by means of a .mp4 video available in the supporting information (file dv5020sup2.mp4).

### 4.2. Seitz symbols for a symmetry operation

**Figure 2:**
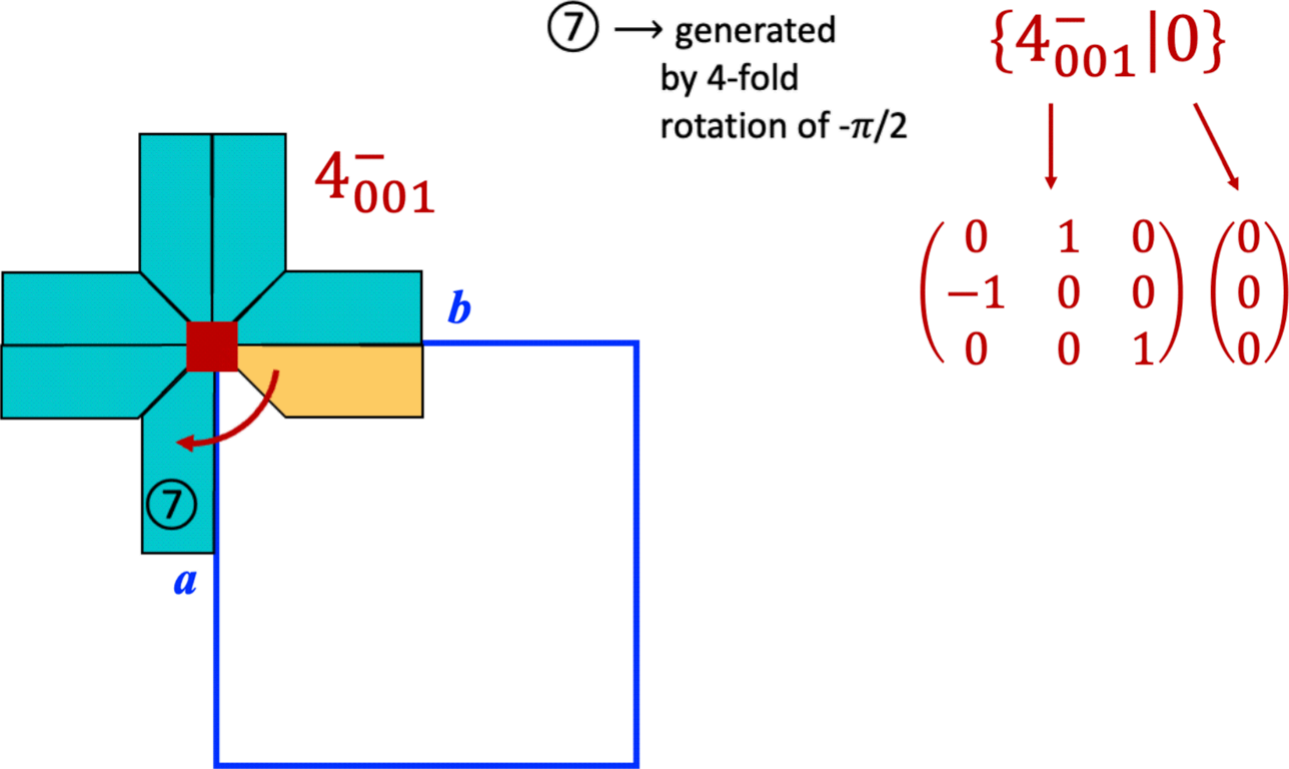
The process of filling the space with the action of symmetry operations on an object (a cross). This screenshot corresponds to one sequence in a supplementary GIF file (dv5020sup3.gif) where the Seitz operator 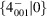 is acting on the asymmetric unit coloured in ochre.

Fig. 2 ▸ is a screenshot of a GIF animation (supplementary file dv5020sup3.gif) developed to illustrate how, by applying the symmetry operations (represented by the Seitz operators), space can be filled. These symmetry operations are always applied to the asymmetric unit starting with the rotational part, with the translation part added at the end. The screenshot displayed in Fig. 2 ▸ was taken just at the time when the Seitz operator 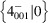 is applied to the asymmetric unit coloured in ochre. As a result of this mapping (movement), the generation of the equivalent region labelled with the number 7 occurs. The rotational and translational parts of the symmetry operation as a matrix and column are indicated in the figure. The complete animation filling a portion of space around the unit cell can be consulted by viewing the supplementary file.

### 4.3. Diagrams for representing general positions and symmetry elements

**Figure 3:**
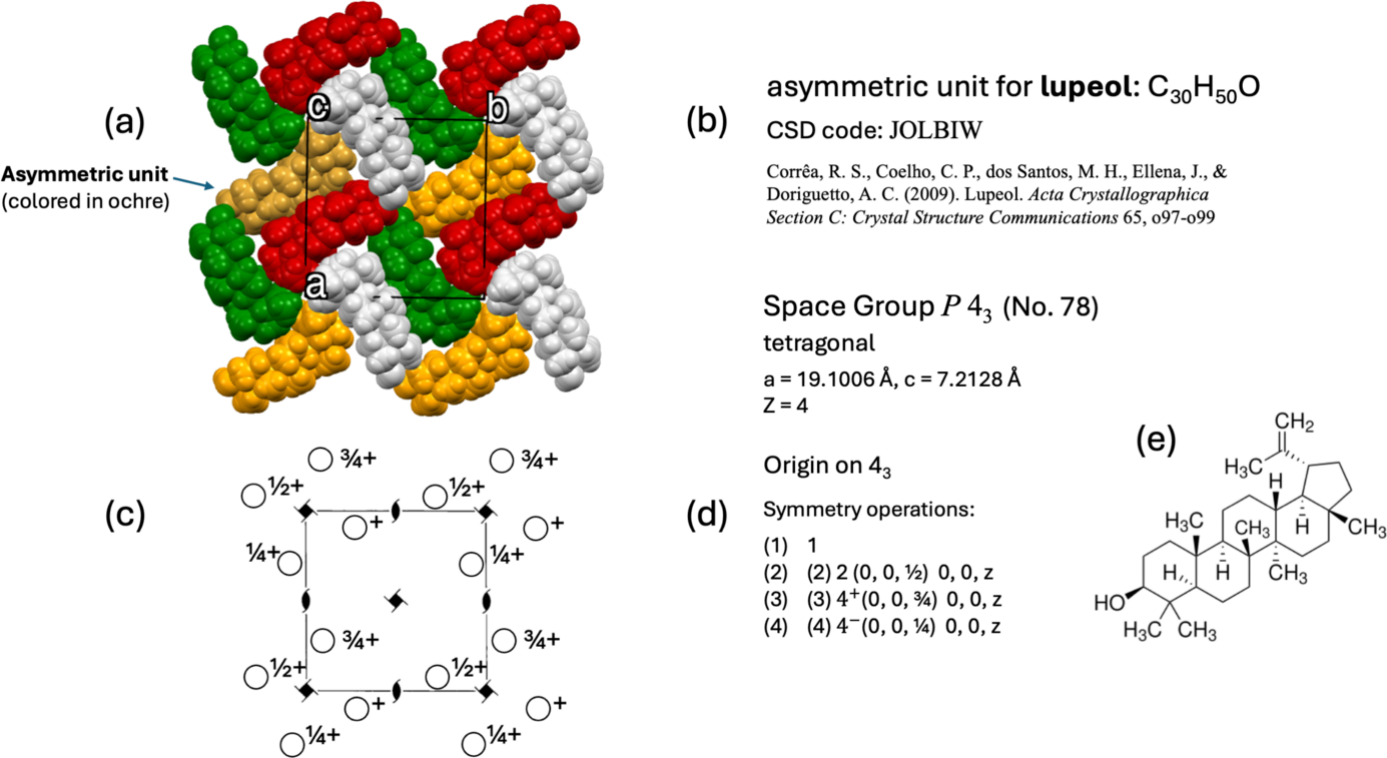
(*a*) The lupeol crystal structure, with the asymmetric unit coloured in ochre. (*b*) Data from Corrêa *et al.* (2009 ▸), CSD refcode JOLBIW. (*c*) A diagram for general positions with the diagram of symmetry elements superimposed. (*d*) The four symmetry operations for the space group *P*4_3_. (*e*) The lupeol chemical structure.

To introduce this topic, the crystal structure of lupeol was selected to illustrate both the diagram of general positions and the diagram representing the symmetry elements. Lupeol is a pentacyclic triterpene and classed as one of the secondary metabolites, considered not to be vital to the organism that produces them; surprisingly, they seem to provide a unique medium for interrelation with the environment (Chappell, 2002 ▸; Gallo & Sarachine, 2009 ▸) and for this characteristic they have been used as botanical origin markers (Lucero-Gómez *et al.*, 2014 ▸). As can be seen in Fig. 3 ▸(*b*), lupeol crystallizes in the tetragonal system, with symmetry described by the space group *P*4_3_ (No. 78). The asymmetric unit consists of one lupeol molecule, C_30_H_50_O, which has 81 atoms with five rings in its molecular structure [Fig. 3 ▸(*e*)]. All these atoms occupy general positions. In fact, this space group has no special positions; none of its symmetry operations can leave a fixed position in space during the mapping. This can only happen when, for example, in a rotation, the position to be mapped lies precisely on the axis of rotation; or in a reflection, the position lies on the plane of reflection; or the position occupies a centre of inversion. In the case of lupeol, the space group *P*4_3_ has no axes of proper rotation, no planes of reflection and no centres of symmetry. Therefore, it cannot have special positions. This space group is a member of just 13 Bieberbach groups that exhibit no special Wyckoff positions. Such groups are called *fixed-point-free space groups* or *Bieberbach groups* and are precisely those groups that may contain glide reflections or screw rotations, but no proper reflections, rotations, inversions or rotoinversions (Souvignier, 2015 ▸).

By applying the four symmetry operations listed in Fig. 3 ▸(*d*), a portion of the space around the unit cell [Fig. 3 ▸(*a*)] has been populated with molecules, where each molecule has been coloured according to the symmetry operation that was applied. The colour has been maintained for each symmetry operation when combined with trans­lations. The action of each symmetry operation on the asymmetric unit for lupeol is represented as a GIF animation in the supplementary file (dv5020sup4.gif). Each mapping movement of the Seitz operator on the asymmetric unit is accompanied by a display of the corresponding general position. Thereby, little by little, the diagram of general positions is completed in the unit cell, reaching the same format as that used in *IT*A. With the help of the diagram for general positions, the diagram of symmetry elements is geometrically elucidated [Fig. 3 ▸(*c*)].

### 4.4. Centred lattices

**Figure 4:**
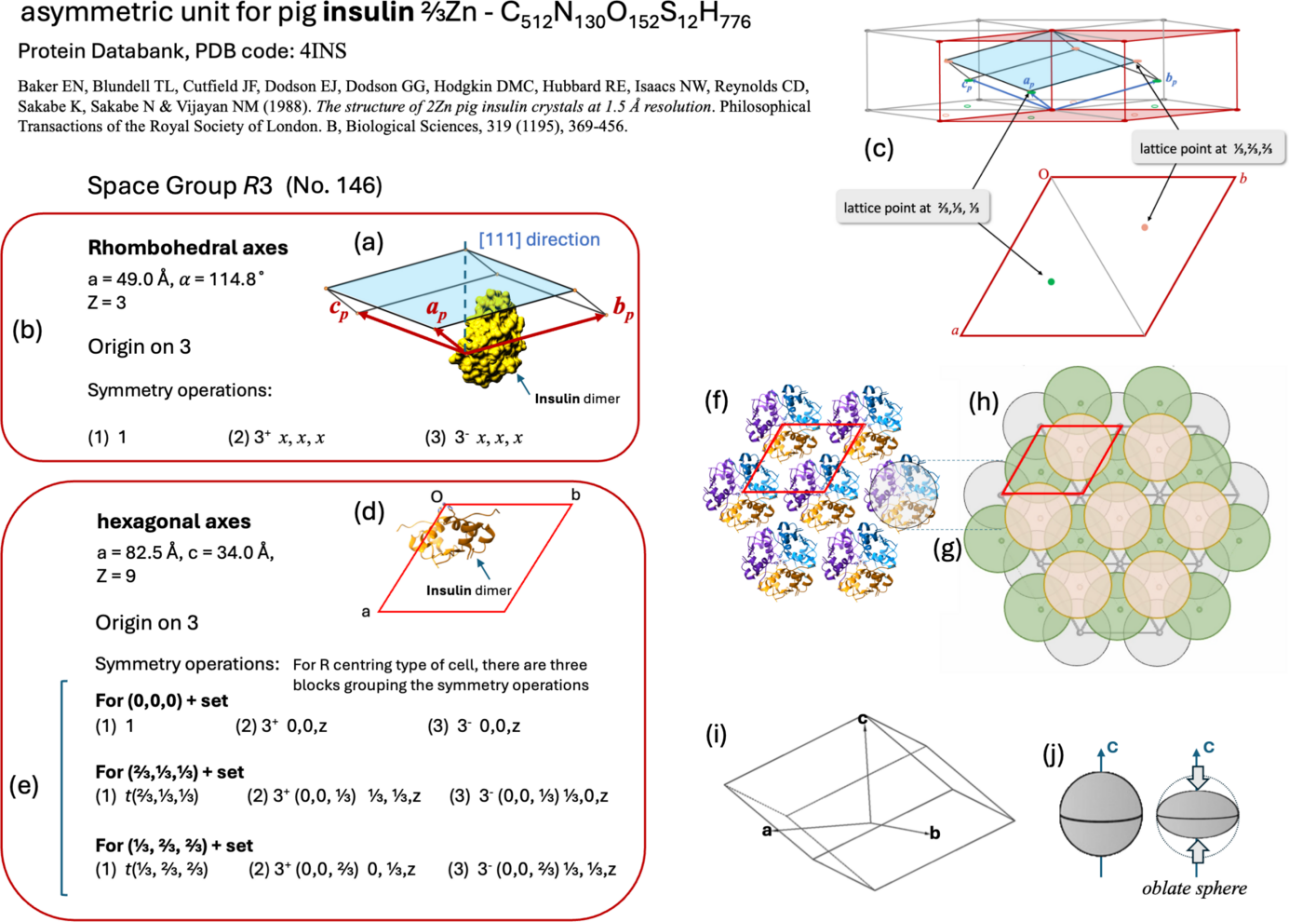
Data for pig insulin according to Baker *et al.* (1988 ▸). (*a*) Asymmetric unit located in the primitive rhombohedral unit cell. (*b*) Symmetry operations for the crystal structure of pig insulin (rhombohedral axes). (*c*) Relation between the descriptions using rhombohedral axes and hexagonal axes. (*d*) Asymmetric unit located in the obverse triple hexagonal cell. (*e*) Symmetry operations for the crystal structure of pig insulin (hexagonal axes). (*f*) Complete fragment of one sheet of packed hexamers of pig insulin. (*g*) One insulin hexamer from panel (*f*), represented as a compact oblate spheroid, constituted of three insulin dimers coordinated around two Zn ions. (*h*) Packing of oblate spheroid insulin hexamers, with layers distinguished by colour. (*i*) Rhombohedral shape for pig insulin crystals. (*j*) Oblate spheroid representation for an insulin hexamer with symmetry axis along **c**.

The crystal structure of pig insulin has two descriptions. One of them is an example of a centred lattice. The crystal structure of 2Zn pig insulin is shown in Fig. 4 ▸, based on data from Baker *et al.* (1988 ▸). It forms rhombohedral crystals that crystallize in the trigonal system, with symmetry described by the space group *R*3 (No. 146) using rhombohedral axes [Figs. 4 ▸(*a*) and 4 ▸(*b*)] or hexagonal axes [Figs. 4 ▸(*d*) and 4 ▸(*e*)]. The relationship between the two descriptions is displayed in Fig. 4 ▸(*c*). If the rhombohedral axis description is used, then three symmetry operations are used [Fig. 4 ▸(*b*)]: (i) identity, (ii) threefold anticlockwise rotation and (iii) threefold clockwise rotation along the [111] direction. They are presented sequentially in a video, including their matrix representations (supplementary file dv5020sup5.mp4). The centred lattice *R* implies the presence of centring translations 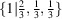 and 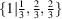 together with {1|0} as symmetry operations [Fig. 4 ▸(*d*)].

In general, the symmetry operations for space groups with centred lattices are represented in *IT*A with the symmetry operations grouped in blocks; the number of blocks is the number of centring translations. The case of insulin is covered in the supplementary file dv5020sup5.mp4. When this video file is executed, the movements produced by the symmetry operations – which always apply to the asymmetric unit – start to be displayed. The demonstration begins with the first block carrying the legend ‘(0,0,0) + set’ as subhead [Fig. 4 ▸(*d*)]. As three symmetry operations are grouped in the first block, *i.e.* (i) identity, (iii) threefold anticlockwise rotation and (iii) threefold clockwise rotation, they are presented sequentially in the video, including their matrix representations. Afterwards, the three symmetry operations of the block are combined with some translations to complete part of ‘the set’. Next, the second block with the subheading ‘For (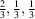) + set’ is presented. It groups the following symmetry operations: (iv) centring translation 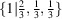, (v) threefold anticlockwise rotation at 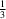, 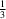, *z* and (vi) threefold clockwise rotation at 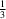, 0, *z*. These symmetry operations result from combining the three symmetry operations of the first block with the centring translation 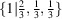. In a similar way, the third block with another three symmetry operations is presented, this time as a result of combining the three symmetry operations of the first block with the centring translation 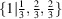.

The asymmetric unit for pig insulin [Figs. 4 ▸(*a*) and 4 ▸(*b*)] consists of a dimer with two insulin molecules. Its chemical formula is ⅔Zn–C_512_N_130_O_152_S_12_H_776_ accompanied by ‘some 283 other atoms, mainly water’ (Baker *et al.*, 1988 ▸). Insulin was chosen to describe symmetry properties here because it shows an asymmetric unit with hundreds of atoms. The video file dv5020sup5.mp4 illustrates how this enormous set of atoms can be represented in the diagram of general positions, which moves according to the action of the symmetry operation. Since the Zn ions are located on the threefold rotation axes, they do not move with the action of the threefold rotation, so they occupy a special position. Additionally, we consider that the video will, in a small way, help in quickly eliminating the idea of seeing lattice points as atomic positions.

### 4.5. Reinterpretation of symmetry elements: relocation, screw axes and glide planes

The combination of rotational symmetry (proper or improper) with translations gives symmetry operations that can be visualized with the symmetry element displaced in space; in other cases, the visualization of the symmetry operation is better perceived as a screw rotation or a glide plane operation. To illustrate this, the crystal structures for ice Ih and aspirin have been selected.

#### 4.5.1. Ice Ih, *P*6_3_*cm* (No. 185)

**Figure 5:**
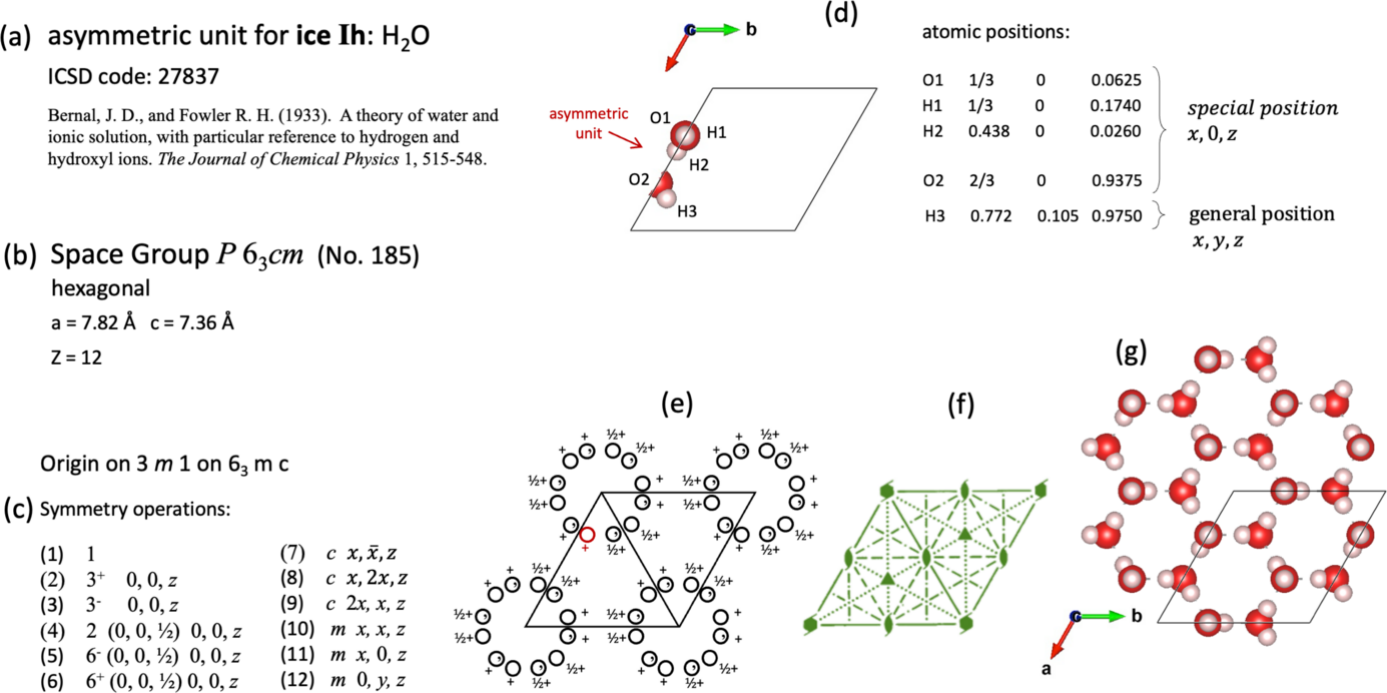
(*a*) Data information for ice Ih from Bernal & Fowler (1933 ▸). (*b*) and (*c*) Space-group symmetry and list of symmetry operations. (*d*) Asymmetric unit for ice Ih. (*e*) Diagram for general positions. (*f*) Diagram for symmetry elements. (*g*) Crystal structure fragment for Ice Ih.

Although ice has been reported to form crystals with many polymorphs, it usually crystallizes developing crystals with hexagonal symmetry in space group *P*6_3_*cm* (No. 185). The asymmetric unit for ice is constituted by five atoms: two oxygen and three hydrogen atoms [Fig. 5 ▸(*d*)].

Since this space group has 12 symmetry operations [Fig. 5 ▸(*c*)], 12 circles symbolizing generated general positions are shown in the unit cell [Fig. 5 ▸(*e*)]. The movements caused by a symmetry operation (supplementary file dv5020sup6.mp4) are executed in the video animation, always starting from a superimposed image in the asymmetric unit which starts to move. The movement stops when the destination of the mapping is reached. At this moment, the corresponding position (as a circle) is shown in the diagram of general positions in the animation. When a symmetry operation is applied, the animation also indicates the corresponding Seitz symbol, as well as the name of the symmetry operation. With this kind of presentation, we have tried to emphasize that the set of atoms that make up the asymmetric unit can be associated with a general position (a circle) as a representative in the diagram of general positions. For those atoms in the asymmetric unit that occupy special positions, the animation shows that they will not move with the action of the symmetry operation. This is exactly the case shown in Fig. 5 ▸(*d*), where all the atoms O1, H1, H2 and O2 of the asymmetric unit for ice lie on a plane of reflection; only atom H3 is on a general position.

The crystal structure of ice is a good example to illustrate the presence of rotation screw axes and glide planes. They can be identified in the list of symmetry operations given in Fig. 5 ▸(*c*). The relocation of symmetry operations because of combining rotations with translations can be observed in the supplementary file dv5020sup6.mp4.

**Figure 6:**
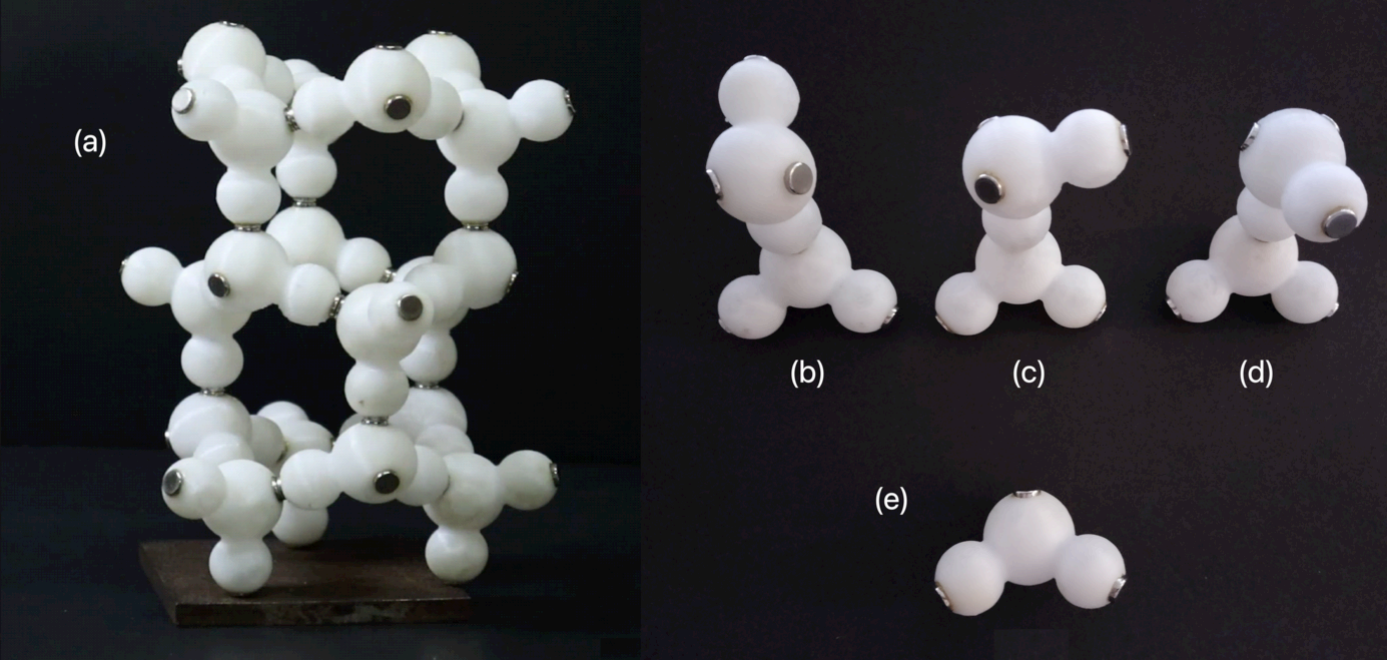
(*a*) Three-dimensional model for the crystal structure of ice. (*b*)–(*d*) Dimers composed of two water molecules. (*b*) The lowest energy equilibrium ‘*trans*’ configuration showing a mirror plane symmetry (Petrenko & Whitworth, 2002 ▸). (*c*) and (*d*) Unstable configurations, where the dipole moments of the two water molecules do not point in such a way that they oppose each other as much as possible. (*e*) Water molecule showing magnets located at the lone-pair positions on the acceptor sites (in the oxygen atom) and on the proton donor sites (the hydrogen atoms).

Two further supplementary materials for the ice crystal structure have been included: dv5020sup7.gif is a GIF animation showing the construction of the ice structure using 3D models, with magnets representing the hydrogen-bond interactions (Fig. 6 ▸), and dv5020sup8.mp4 is a video showing how the models are manipulated by children and a teenage girl to build the ice structure. Prior to practice, they were asked to perceive and verify the forces of attraction and repulsion occurring when the magnets were brought together (*i.e.* orienting magnets exhibiting their north pole at hydrogen atoms and others exhibiting their south pole at oxygen acceptor sites). In this context, they were asked to think about the arrangement they would assume would be more satisfactory for a water dimer [Figs. 6 ▸(*b*), 6 ▸(*c*) and 6 ▸(*d*)].

#### 4.5.2. Aspirin, *P*2_1_/*c*1 (No. 14)

**Figure 7:**
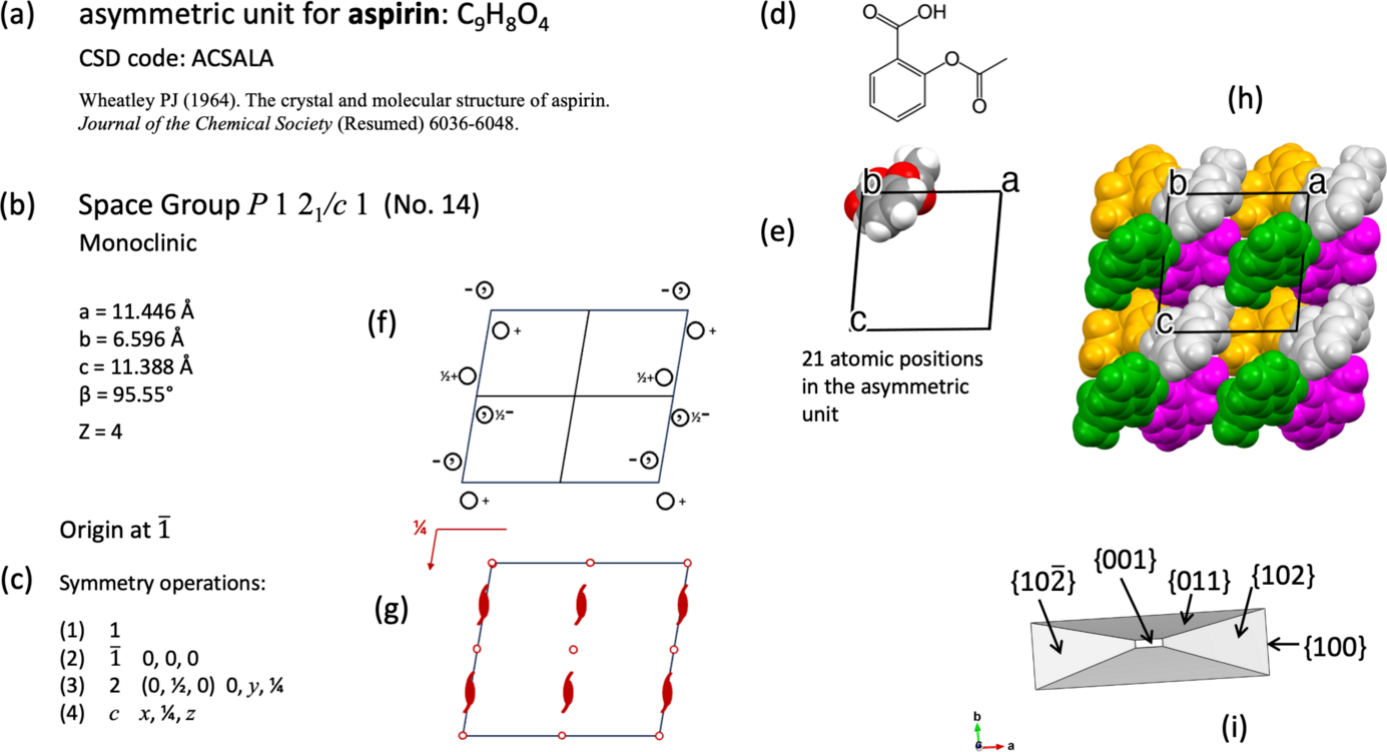
(*a*) Data for aspirin from Wheatley (1964 ▸). (*b*) Space group and crystallographic data. (*c*) Symmetry operations. (*d*) Molecular formula. (*e*) Asymmetric unit. (*f*) Diagram for general positions. (*g*) Diagram for symmetry elements. (*h*) Fragment for aspirin crystal structure. (*i*) Crystal forms observed during crystal growth of aspirin (video can be seen at the end of supplementary file dv5020sup9.mp4).

Aspirin crystallizes in the monoclinic system with space group 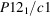 (No. 14). It represents another example where screw axes and glide planes can be found as symmetry operations [Figs. 7 ▸(*b*) and 7(*c*)]. The asymmetric unit consists of one aspirin molecule [Figs. 7 ▸(*d*) and 7(*e*)] with chemical formula C_9_H_8_O_4_, with all 21 atoms occupying general positions. Inversion centres are the unique sites for special positions for this space group.

**Figure 8:**
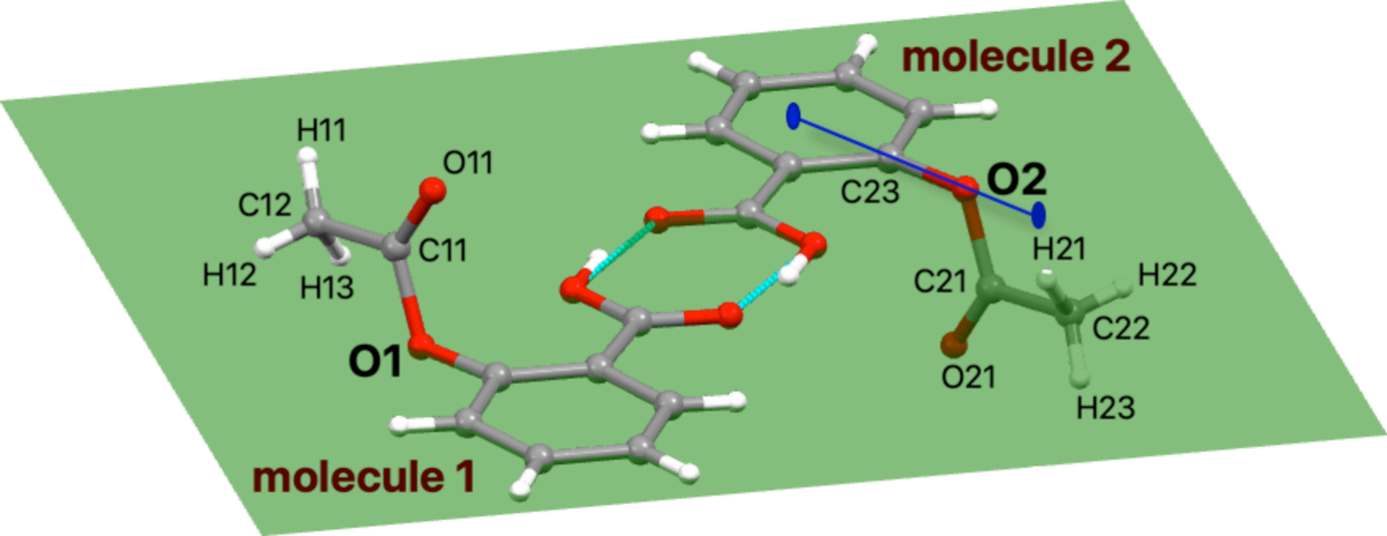
Two molecules of aspirin, molecule 1 and molecule 2, are related by an inversion centre. These two molecules are not enantiomers, since the mirror image of molecule 1 (*i.e.* molecule 2) can be superimposed on molecule 1 by rotating the fragment C21/C22/O21/H21/H22/H23 with respect to a twofold axis defined by the line C23–O2, indicated by a blue line.

When the symmetry operations listed in Fig. 7 ▸(*c*) are combined with translations, the portion of the crystal structure shown in Fig. 7 ▸(*h*) is obtained. The sequence of symmetry map­pings is shown in a supplementary video file dv5020sup9.mp4. The procedure followed in the video for exhibiting each symmetry mapping in aspirin was as follows. The Seitz symbol that represents the symmetry operation and its name are displayed. Thereafter, an image of a circle appears superimposed on the general position that corresponds to the asymmetric unit; it then starts moving until it reaches the destination specified by the symmetry operation. At the end, the circle becomes frozen in the diagram of symmetry operations. At this moment, an image of the set of atoms generated by symmetry is displayed and added to the image of the crystal structure. The aspirin molecule has no enantiomers, so it is not a chiral molecule. However, the asymmetric unit in the crystal structure of aspirin is related to another molecule by means of an inversion centre (Fig. 8 ▸). A video of aspirin crystals growing has been added at the end of the supplementary file dv5020sup9.mp4.

### 4.6. Coset decomposition of a space group

**Figure 9:**
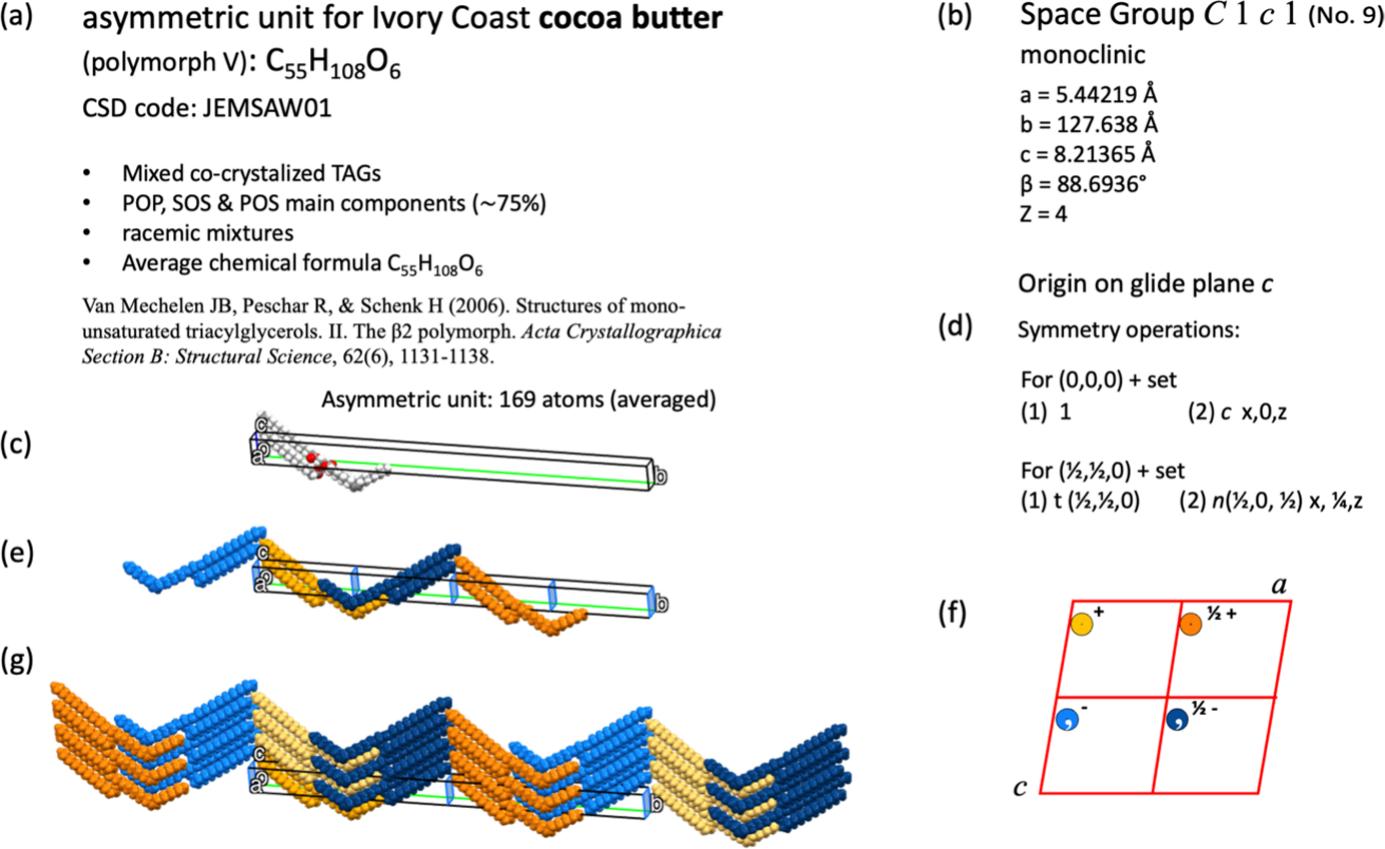
(*a*) Information for the asymmetric unit of polymorph V of cocoa butter. (*b*) Crystallographic data and space group. (*c*) Asymmetric unit. (*d*) Symmetry operations. (*e*) Molecules generated by the four symmetry operations and (*f*) the corresponding diagram for general positions. (*g*) Crystal structure.

Fig. 9 ▸ shows form V of cocoa butter (Mechelen* et al.*, 2006 ▸), which crystallizes in the monoclinic system with space group *C*1*c*1 (No. 9). The asymmetric unit is complicated: it consists of one molecule of tri­acyl­glycerol (TAG), which can be 1,3-di­palmitoyl-2-oleoylglycerol (POP), 1,3-distearoyl-2-oleoylglycerol (SOS) or 1-palmitoyl-2-oleoyl-3-stearoylglycerol (POS). These are the main components of TAGs in cocoa butter (∼75%); each TAG appears as a racemic mixture co-crystallized with the other TAGs (enantiomers are present as a racemic mixture in the components of cocoa butter).

**Figure 10:**
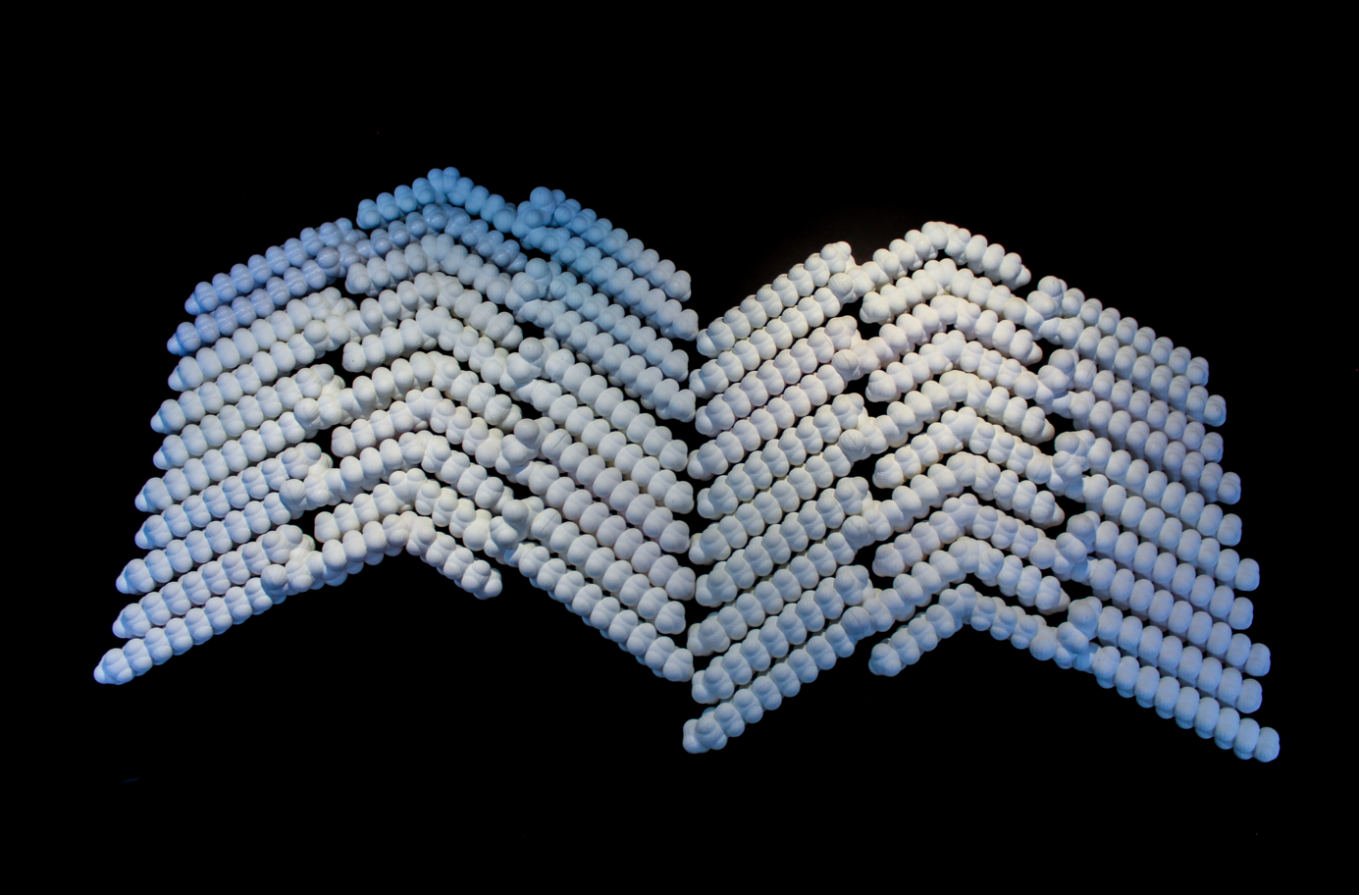
Three-dimensional models representing the arrangement for cocoa butter (form V).

The crystalline structure for cocoa butter has an asymmetric unit with average chemical formula C_55_H_108_O_6_, with all 169 atoms occupying general positions (this space group does not have special positions). The sequence of symmetry operations listed in Fig. 9 ▸(*d*) is shown in the supplementary video file dv5020sup10.mp4. The procedure was as follows. The Seitz symbol that represents the symmetry operation and its name are displayed. Hereafter, an image of the asymmetric unit appears superimposed on the asymmetric unit in the crystal structure, and then starts moving until it reaches the destination specified by the symmetry operation. At the end, a circle appears as a general position in the diagram of general positions. The application of the four symmetry operations listed in Fig. 9 ▸(*d*) generates four imaged molecules which are displayed in different colours in the animations [Fig. 9 ▸(*e*)]. The combination of these four symmetry operations with some translations allows the crystal structure to be represented [Fig. 9 ▸(*g*)]. In this case, the mappings of each symmetry operation combined with some translations are represented using one characteristic colour. If the four symmetry operations listed in Fig. 9 ▸(*d*) are referred to as *coset representatives*, the space group 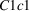 can be represented as a *left coset* decomposition with respect to its translation subgroup 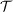 as follows:
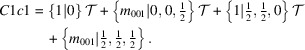
This representation is illustrated at the end of the video supplementary file dv5020sup10.mp4. Three-dimensional models to build form V of the crystal structure of cocoa butter have been used in our laboratory (Fig. 10 ▸).

## 5. Crystal growth of chromium potassium alum

**Figure 11:**
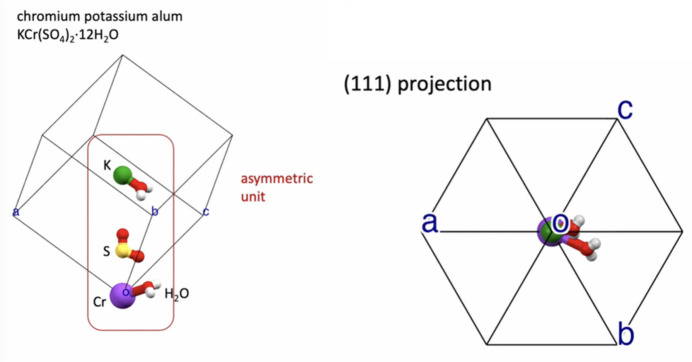
(Left) Asymmetric unit for chromium potassium alum crystal structure. (Right) Asymmetric unit viewed on a (111) projection. Data from Bacon & Gardner (1958 ▸), ICSD code 38232. These images are screenshots taken from the supplementary file dv5020sup11.mp4.

**Figure 12:**
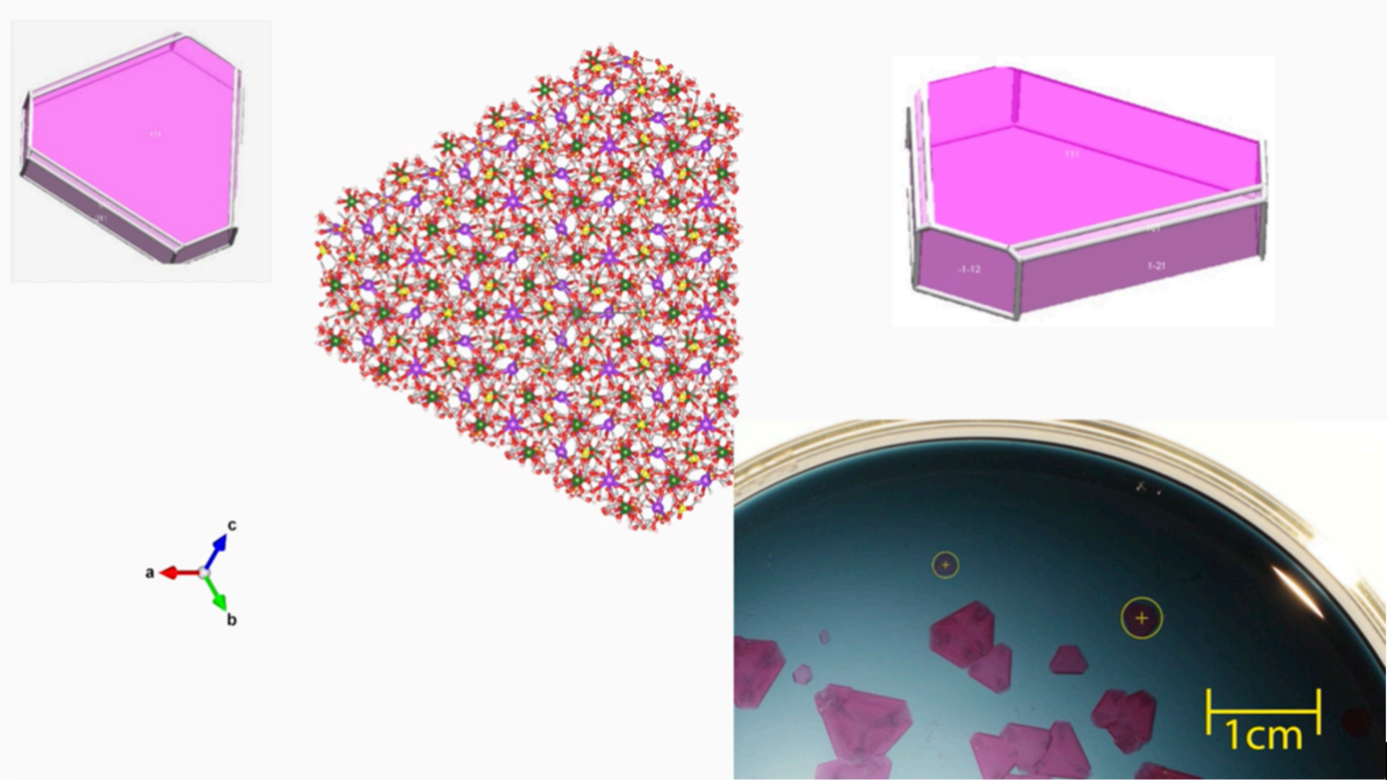
The way in which the Seitz operators are applied to fill the 3D space, with a macroscopic version that can clearly be seen in the videos of crystals growing.

Chromium potassium alum, KCr(SO_4_)_2_·12H_2_O, is relatively easy to crystallize in an ordinary laboratory. The crystals obtained have symmetry belonging to the cubic system, with space group 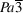 (No. 205). The asymmetric unit (Fig. 11 ▸) consists of ten atoms. When the video file dv5020sup11.mp4 is run, the animation starts off showing the asymmetric unit making a rotation, to exhibit clearly the positions of all the atoms of the asymmetric unit. This step is announced by showing the Seitz symbol and name of the identity operation {1|0}. With each of the next few symmetry operations, sequentially applied, the subset of atoms generated by the new symmetry operation appear coloured in black. The animation carries out this action executing a rotation, keeping the atoms generated by symmetry in black. The purpose of this is to show clearly the arrangement of all the atoms under the action of the symmetry operation. When the next symmetry operation starts, the set of atoms that were coloured in black change their colours according to each chemical species. In this way, the 3D space is filled little by little, with the intention of mimicking macroscopic crystal growth. That is to say, the way in which the Seitz operators are applied to fill the 3D space is related to a macroscopic version that can be clearly attested by the videos of crystals of chromium potassium alum growing (Fig. 12 ▸). The corresponding video file is included at the end of the supplementary file dv5020sup11.mp4.

## 6. Discussion and conclusions

In their process of learning, many of our students find it difficult to identify regions that are symmetrically equivalent to each other and therefore do not easily find the presence of certain symmetry operations and symmetry elements in space. In general, the analysis of crystal structure fragments that are related to each other by their space-group symmetry operations is a subject that has been of interest when reviewing conference presentations, theses and manuscripts for refereed publications. The need to improve the teaching material offered to students inside and outside the classroom was stimulated by the doubts that arose when consulting *IT*A for the understanding of certain crystal structures published in the literature. For numerous students – including those from areas such as medicine, dentistry, biology, anthropology, architecture *etc.* – the criteria to select the views that clearly show how the atoms (or molecules) are arranged in a crystalline solid have acted as a bridge to attract these people to discuss the crystal structure in a fascinating manner, since they see the necessity of putting the crystal structure into context for the practical use they desire.

During the COVID-19 pandemic, we were forced to interact with students through digital and audiovisual means. Quickly, the creation of animations and videos represented an attractive solution for students when studying *IT*A and some crystal structures. The task was to create movement to the diagrams for general positions, in combination with crystal structures and crystal growth experiences filmed on video. As a result, many students reacted favourably when they had the chance to see the short briefing capsules concerning crystals such as insulin, aspirin, cocoa butter or ice, among others.

At the time of writing of this manuscript, it is not clear to us what effect the use of these capsules will have on the learning of the topics addressed. However, for the evaluation and improvement of the short briefing capsules, we will take into account the questions raised by He (2020 ▸) concerning the problems of using animations: (i) sequential playback makes it difficult to compare and contrast the information contained; (ii) the amount of information to be processed is demanding when simultaneous changes have to be perceived and understood; (iii) the transience of the animation increases the burden for learners to internalize information extracted from the visual representation; (iv) the split attention effect within the representation also leads to problems for perception, as full attention to one part of the screen would lead to neglect of information in other regions.

Basically, we are faced with the problem of the sensation and perception of symmetry. Through the visual sense we have tried to help the perception of symmetry: we can see but not necessarily realize the symmetrical quality of objects. Sensations and perceptions are remarkably dissimilar (Cohen, 1969 ▸).

The information contained in this article is intended to help those who wish to learn how symmetry operations work in a very visual way. It is possible that, with this procedure, details can be observed that it would otherwise not have been possible to pay attention to. Thinking very favourably, with the increased visual perception of the action of the symmetry operations, perhaps it can be a source of inspiration for new ideas or new perceptions. The information presented in the *Introduction*[Sec sec1] to this paper shows that the structural models and other resources proved to be of great benefit to the understanding of crystalline arrangements.

Finally, our experience tries to contribute to reverse the results of the OECD PISA tests in Mexico (https://www.oecd.org/en/publications/pisa-2022-results-volume-i_53f23881-en.html). They showed an educational lag, mainly in science and mathematics, as Mexican students’ scores are below the OECD average, suggesting a limited ability to apply scientific knowledge in a variety of situations. The reasons are diverse, but in the experience of pre-school and primary school teachers, students show a great interest in these areas at these stages, but interest changes in secondary and high school. They find it difficult and many of them do not pass, mainly because they consider it difficult and the way they are taught does not relate to their daily lives, so they do not find it useful to learn it. However, the experiences we have had with primary and high school students working with the 3D models for the ice structure give us some motivating hope. We also hope to contribute to the task of eliminating the factors that have caused crystals and the science of crystallography to be omitted or relegated as optional subjects in educational curricula (Murray *et al.*, 2024 ▸).

## Supplementary Material

List of supporting materials. DOI: 10.1107/S1600576724008872/dv5020sup1.pdf

Video 1. DOI: 10.1107/S1600576724008872/dv5020sup2.mp4

GIF 1. DOI: 10.1107/S1600576724008872/dv5020sup3.gif

GIF 2. DOI: 10.1107/S1600576724008872/dv5020sup4.gif

Video 2. DOI: 10.1107/S1600576724008872/dv5020sup5.mp4

Video 3. DOI: 10.1107/S1600576724008872/dv5020sup6.mp4

Video 4. DOI: 10.1107/S1600576724008872/dv5020sup7.gif

Video 5. DOI: 10.1107/S1600576724008872/dv5020sup8.mp4

Video 6. DOI: 10.1107/S1600576724008872/dv5020sup9.mp4

Video 7. DOI: 10.1107/S1600576724008872/dv5020sup10.mp4

GIF 3. DOI: 10.1107/S1600576724008872/dv5020sup11.mp4

## Figures and Tables

**Table 1:** Crystal structures considered in the animations, videos and 3D models

Name	Chemical formula	Crystal system	Space group	CIF file
Lupeol	C_30_H_50_O	Tetragonal	*P*4_3_ (No. 78)	JOLBIW^(*a*)^
Insulin	Zn–C_512_N_130_O_152_S_12_H_776_	Trigonal	*R*3 (No. 146)	4ins ^(*b*)^
Ice (Ih)	H_2_O	Hexagonal	*P*6_3_*cm* (No. 185)	27837^(*c*)^
Aspirin	C_9_H_8_O_4_	Monoclinic	*P*12_1_/*c*1 (No. 14)	ACSALA^(*a*)^
Cocoa butter (polymorph V)	C_55_H_108_O_6_	Monoclinic	*C*1*c*1 (No. 9)	JEMSAW01^(*a*)^
Chromium potassium alum	KCr(SO_4_)_2_·12H_2_O	Cubic	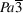 (No. 205)	38232^(*c*)^

Sources for the CIF files: (*a*) Cambridge Structural Database (CSD; https://www.ccdc.cam.ac.uk/solutions/software/csd/), (*b*) Protein Data Bank (PDB; https://www.rcsb.org/), (*c*) Inorganic Crystal Structure Database (ICSD; https://icsd.fiz-karlsruhe.de/index.xhtml).

**Table 2:** Short briefing capsules available in the supporting information The first column refers to subsections of this article. The final column specifies the running times of the capsules (min:s) and the corresponding names of the supplementary files.

Subsection	Skill and level	Running time
4.1	Ability to obtain the matrix representation of rotational symmetry operations. *Basic level: linear algebra and crystallographic notation required.*	4:47 (dv5020sup2.mp4)
4.2	Ability to use Seitz symbols and the application of symmetry operations using matrix notation. *Basic level: linear algebra, crystallography and its notation required.*	9:27 (dv5020sup3.gif)
4.3	Practice on diagrams to represent general positions and to display symmetry elements in the lupeol crystal structure. *Intermediate level: elementary crystallography and its notation required.*	1:35 (dv5020sup4.gif)
4.4	Practice on a centred lattice in the trigonal system: space group *R*3 for the case of insulin. Descriptions using the rhombohedral space group with a primitive rhombohedral unit cell based on rhombohedral axes and using an obverse triple hexagonal cell based on hexagonal axes. *Advanced level: crystallography and its notation required.*	31:29 (dv5020sup5.mp4)
4.5.1	Ability to reinterpret symmetry elements and relocation, as well as diverse symmetry operations in the crystal structure of ice. *Intermediate level: elementary crystallography and its notation required.*	13:19 (dv5020sup6.mp4)
		(dv5020sup7.gif)
		3:47 (dv5020sup8.mp4)
4.5.2	Ability to reinterpret symmetry elements and relocation, as well as diverse symmetry operations in the crystal structure of aspirin. *Intermediate level: elementary crystallography and its notation required.*	7:26 (dv5020sup9.mp4)
4.6	Practice on coset decomposition of a space group in cocoa butter (form V). *Intermediate level: crystallography and its notation required.*	4:03 (dv5020sup10.mp4)
5	Practice on symmetry operations and crystal growth in chromium potassium alum. *Intermediate level: crystallography and its notation required.*	2:12 (dv5020sup11.mp4)
