# Supplementary material for: Animations, videos and 3D models for teaching space-group symmetry
Source: J Appl Crystallogr. 2024 Oct 16;57(Pt 6):1966–77. doi: 10.1107/S1600576724008872 (PMC11611277; doi:10.1107/S1600576724008872)
Supplement: Supplementary file 1 [file j-57-01966-sup1.pdf]

JOURNAL OF
APPLIED
CRYSTALLOGRAPHY

Volume 57 (2024)

Supporting information for article:

Animations, videos and 3D models for teaching space-group symmetry

Lauro Bucio, Rosario Moreno-Tovar, Edilberto Hernández-Juárez, Andrea S. Sandoval-Santiago, Nerith R. Elejalde-Cadena, Andrés Bucio, Moises Falcón-Moreno and Ivonne Rosales-Chávez

dv5020sup1.pdf This list of video files.

dv5020sup2.mp4 Matrix representation for rotational symmetry operations

The matrix representation for five isometries $\alpha = 2_{001}, 4^{-}_{001}, \bar{6}^{+}_{001}, m_{210},$ and $\bar{3}^{+}_{111}$ is geometrically rationalized by means of an mp4 video.

dv5020sup3.gif Seitz symbols for a symmetry operation

The complete actions of all symmetry operations of a cross with Seitz symbols and their correspondent matrix representation.

dv5020sup4.gif Diagrams for representing general positions and symmetry elements for lupeol

The crystalline structure of lupeol is used to illustrate the diagrams for general positions, and the diagrams for symmetry elements according to ITA.

dv5020sup5.mp4 Symmetry for 2Zn insulin crystalline structure: a case for centred lattice

The mapping using the Seitz operators defined in the space group for insulin can be carefully noted running this video. The matrix representation of symmetry operations has been included in the animation for the two descriptions using a primitive rhombohedral cell with rhombohedral axes, and an obverse triple hexagonal cell with hexagonal axes.

dv5020sup6.mp4 Crystal symmetry for ice: relocation of symmetry elements, screw axes and glide planes

The mapping using the Seitz operators defined in the space group for ice can be carefully noted running the animation.

dv5020sup7.gif Building a model for ice using 3D models

Video showing the building of the ice structure using 3D models with magnets representing the hydrogen-bond interactions in ice (Figure 6).

dv5020sup8.mp4 Building a model for ice by a child using 3D models

Video showing the building of the ice structure using 3D models with magnets by the children and a teenage girl.

dv5020sup9.mp4 Crystal symmetry for aspirin

A video showing the process of mapping by each symmetry operation of the space group for aspirin.

dv5020sup10.mp4 Crystal symmetry for cocoa butter (form V)

A video showing the process of mapping by each symmetry operation of the space group in cocoa butter.

dv5020sup11.mp4 Crystal growth in chromium potassium alum

The asymmetric unit (Figure 11) is constituted of ten atoms. The way in which the Seitz operators are applied to fill the three-dimensional space reaches a macroscopic version that can be clearly attested by the filmed videos of crystals growing.
