# Supplementary figures and images for: Animations, videos and 3D models for teaching space-group symmetry
Source: J Appl Crystallogr. 2024 Oct 16;57(Pt 6):1966–77. doi: 10.1107/S1600576724008872 (PMC11611277; doi:10.1107/S1600576724008872)

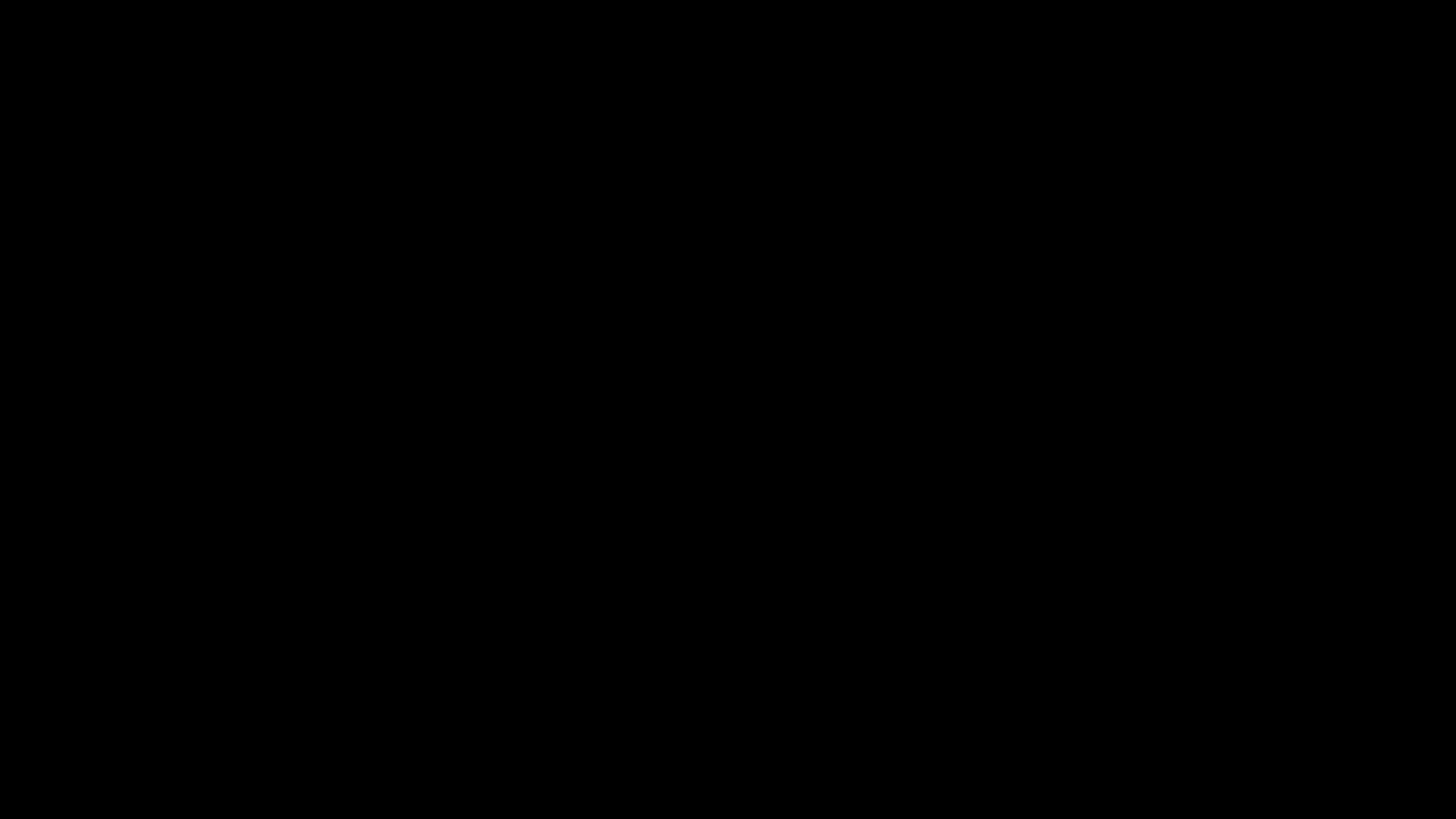

Supplement: Supplementary file 3 [file j-57-01966-sup3.gif]

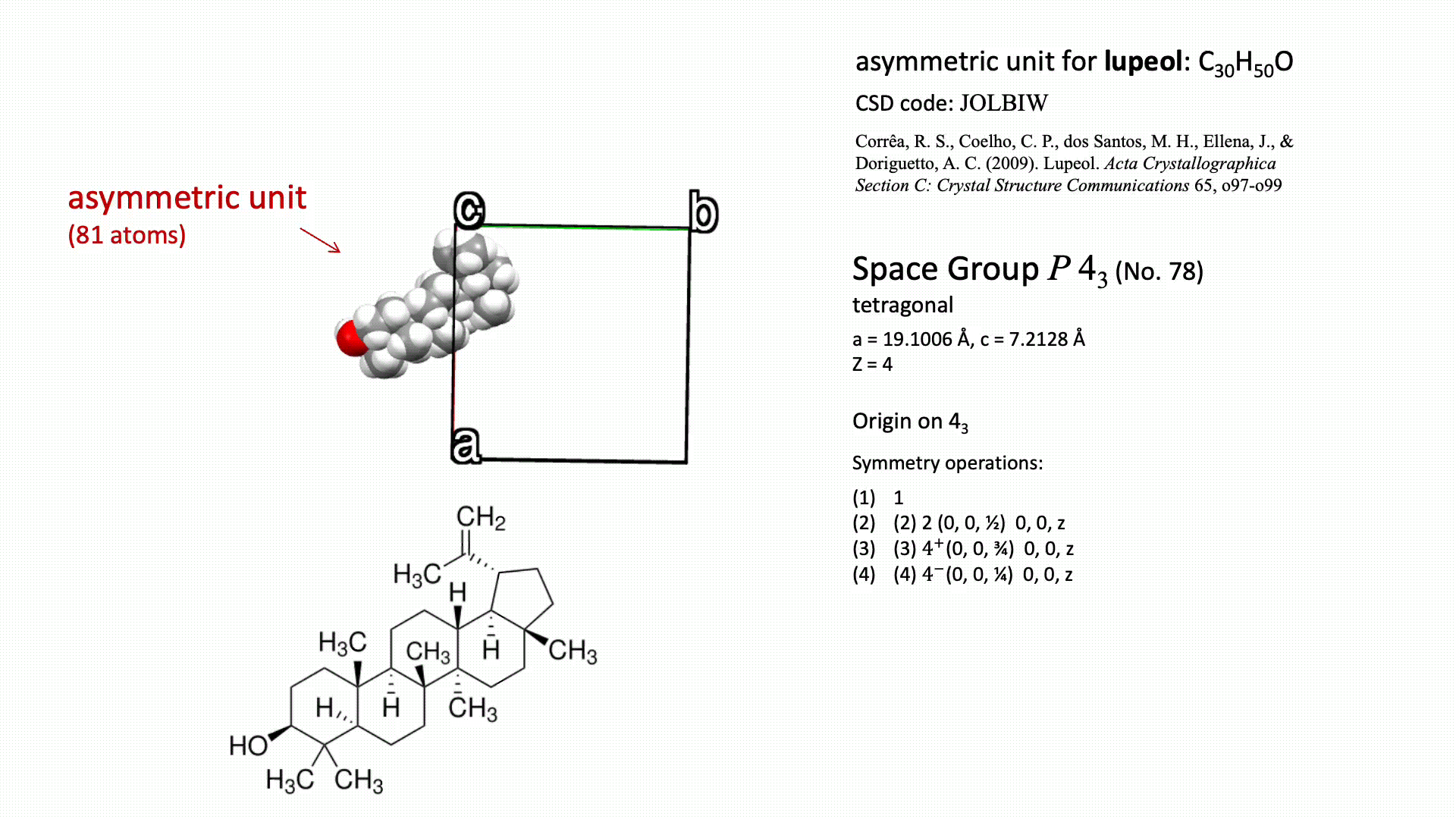

Supplement: Supplementary file 4 [file j-57-01966-sup4.gif]
